# External Validation of a Breath-Based Prediction Model for Malignant Pleural Mesothelioma

**DOI:** 10.3390/cancers14133182

**Published:** 2022-06-29

**Authors:** Eline Janssens, Eline Schillebeeckx, Kathleen Zwijsen, Jo Raskin, Joris Van Cleemput, Veerle F. Surmont, Kristiaan Nackaerts, Elly Marcq, Jan P. van Meerbeeck, Kevin Lamote

**Affiliations:** 1 Laboratory of Experimental Medicine and Pediatrics, Infla-Med Center of Excellence, University of Antwerp, 2610 Antwerp, Belgium; eline.janssens2@uantwerpen.be (E.J.); eline.schillebeeckx@uantwerpen.be (E.S.); kathleen.zwijsen@uantwerpen.be (K.Z.); jan.vanmeerbeeck@uza.be (J.P.v.M.); 2 VIB-UGent Center for Medical Biotechnology, 9000 Ghent, Belgium; 3 Department of Pulmonology & Thoracic Oncology, Antwerp University Hospital, 2650 Edegem, Belgium; jo.raskin@uza.be; 4 Occupational Health Service, Eternit N.V., 1880 Kapelle-op-den-Bos, Belgium; joris.van.cleemput@eternit.be; 5 Department of Respiratory Medicine, Ghent University Hospital, 9000 Ghent, Belgium; veerle.surmont@uzgent.be; 6 Department of Respiratory Medicine, University Hospital Gasthuisberg, 3000 Leuven, Belgium; kristiaan.nackaerts@uzleuven.be; 7 Center for Oncological Research (CORE), Integrated Personalized and Precision Oncology Network (IPPON), University of Antwerp, 2610 Antwerp, Belgium; elly.marcq@uantwerpen.be; 8 Department of Internal Medicine and Pediatrics, Ghent University, 9000 Ghent, Belgium

**Keywords:** pleural mesothelioma, biomarkers, volatile organic compounds, early detection, asbestos

## Abstract

**Simple Summary:**

Malignant pleural mesothelioma (MPM) is an incurable asbestos-related thoracic cancer for which early-stage diagnosis remains a major challenge. Volatile organic compounds (VOCs), which are metabolites present in exhaled breath, have proven to be promising non-invasive biomarkers for MPM. However, without the necessary validation in an independent group of individuals, clinical implementation is hampered. Therefore, we performed external validation of a VOC-based prediction model for MPM, which initially revealed a poor performance and thus poor generalisability of the model. However, subsequent updating of the model improved its performance in the validation cohort, resulting in a more generalisable model with a screening potential, which could significantly impact MPM management.

**Abstract:**

During the past decade, volatile organic compounds (VOCs) in exhaled breath have emerged as promising biomarkers for malignant pleural mesothelioma (MPM). However, as these biomarkers lack external validation, no breath test for MPM has been implemented in clinical practice. To address this issue, we performed the first external validation of a VOC-based prediction model for MPM. The external validation cohort was prospectively recruited, consisting of 47 MPM patients and 76 asbestos-exposed (AEx) controls. The predictive performance of the previously developed model was assessed by determining the degree of agreement between the predicted and actual outcome of the participants (patient/control). Additionally, to optimise the performance, the model was updated by refitting it to the validation cohort. External validation revealed a poor performance of the original model as the accuracy was estimated at only 41%, indicating poor generalisability. However, subsequent updating of the model improved the differentiation between MPM patients and AEx controls significantly (73% accuracy, 92% sensitivity, and 92% negative predictive value), substantiating the validity of the original predictors. This updated model will be more generalisable to the target population and exhibits key characteristics of a potential screening test for MPM, which could significantly impact MPM management.

## 1. Introduction

Malignant pleural mesothelioma (MPM) is an aggressive, incurable thoracic cancer that is strongly associated with asbestos exposure. Despite recent advances in treatment, the five-year survival rate remains only 5 to 10% [1]. It is believed that screening and early detection could reduce mortality, which prompted an ongoing search for MPM biomarkers more than 30 years ago [2,3]. An emerging field of research is the analysis of volatile organic compounds (VOCs) in exhaled breath, referred to as breathomics, which has been extensively studied as a non-invasive approach for the diagnosis of a range of diseases including MPM [4,5,6]. VOCs are considered suitable biomarker candidates as they reflect (patho)physiological processes in the human body because they are (by)products of the cellular metabolism. After entering the bloodstream and circulating to the lungs, these volatile compounds diffuse across the alveolar membrane to eventually be exhaled through breath [4].

Different promising VOC-based prediction models for MPM have been proposed over the years, but none of these have been implemented in clinical practice due to a lack of external validation [7,8,9]. In biomarker development, confirmation of the performance in an independent set of participants (different time and/or location) is compulsory to externally validate the promising findings of the discovery phase. This is essential to evaluate the generalisability of the developed models, ensuring reliable and reproducible predictions [10]. Although external validation in the metabolomics field is highly recommended, many studies fail to perform this and only report internal validation results, which tend to be overoptimistic; so, reproducibility remains a major issue in breath research [11].

Previously, our research group determined the breath VOC profiles of MPM patients and asbestos-exposed (AEx) controls in an initial discovery study using ion mobility spectrometry (IMS) and reported differentiation between both groups with 85% accuracy [12]. To address the lack of validation studies in (MPM) breath research, here, we now performed external validation of this prediction model for MPM in an independent group of individuals, sampled several years after the initial discovery study. Additionally, to further optimise the model’s performance, the model was updated by refitting it to the validation cohort using the predictor variables of the original model as input features. The TRIPOD guidelines for good reporting of studies validating multivariate prediction models were followed [13].

## 2. Materials and Methods

### 2.1. Study Design and Population

A multicentre, cross-sectional, case-control study was set up to recruit the external validation cohort. The study was approved by the ethics committee of the Antwerp University Hospital (Belgian registration number B300201837007) and was conducted in accordance with the Helsinki Convention. Participants were recruited from October 2018 to November 2021. MPM patients were randomly included after referral through the Thoracic Oncology department of the Antwerp University Hospital (Belgium). MPM diagnosis was histologically confirmed, and patients were treatment-naïve at the time of participation. At-risk controls with a known history of asbestos exposure (AEx), both asymptomatic individuals and patients with benign asbestos-related diseases (pleural plaques, asbestosis, and/or pleuritis), were recruited through the occupational health departments of two companies that used asbestos until 1997, and through an online advertisement on the website of the Antwerp University Hospital. Upon inclusion, participants gave written informed consent and completed two questionnaires to check if the inclusion criteria were met and to collect data about their demographics and asbestos exposure history. None of the participants had taken part in the initial discovery study.

### 2.2. Exhaled Breath Sampling and Analysis Procedure

Participants were asked not to eat, drink, or smoke at least two hours prior to sampling. Breath sampling and analysis were carried out using a multicapillary column/ion mobility spectrometer (MCC/IMS; BioScout, B&S Analytik, Dortmund, Germany) with an integrated breath sampler (SpiroScout, Ganshorn Medizin Electronic, Niederlauer, Germany), according to a previously validated protocol [12,14]. An additional viral filter was placed before the inlet of the MCC/IMS device to protect the participants from potential cross-contamination of SARS-CoV-2 during the COVID-19 pandemic. After every breath sample, a background sample was taken by sampling 10 mL of room air. To minimise external contamination, disposable mouthpieces and filters were used, and to remove any potential contaminants, the MCC/IMS device was flushed with humid air between sampling of different participants.

To assess any potential effect of the additional viral filter on the VOC measurements, three test samples with a viral filter and three test samples without a viral filter were obtained from a healthy subject.

### 2.3. Data Processing and Statistical Analysis

#### 2.3.1. Data Pre-Processing

The raw MCC/IMS data consist of chromatograms, visualising individual VOCs separated by their retention time (RT) and inverse reduced ion mobility (1/K_0_). The software VisualNow (B&S Analytik, Dortmund, Germany) was used to pre-process the raw data by (1) chromatogram alignment, (2) baseline correction, (3) normalisation to reactant ion peak (RIP), (4) compensation for RIP-tailing, and (5) smoothening. After pre-processing, VOCs were manually selected and analysed by an analysis expert blinded to the patient outcome, resulting in a peak intensity for each VOC in each sample. To reduce the risk of interference from potential confounding factors from ambient air, the alveolar gradient was determined for each selected VOC by subtracting the peak intensity in the corresponding background sample from the peak intensity in the breath sample. Those alveolar gradient values were used as predictor variables in further statistical analysis.

The data of the six test samples were used to assess any potential effect of the additional viral filter on the VOCs analysed in this study using the paired Wilcoxon signed rank test.

#### 2.3.2. Model Validation

The model characteristics reported in the initial discovery study were obtained by performing least absolute shrinkage and selection operator (lasso) regression with leave-one-out cross-validation (LOOCV) [12]. The VOCs that were selected in at least 80% of the folds of the LOOCV were considered the most important variables in the differentiation between MPM patients and AEx controls (with and without benign asbestos-related diseases). To extract the final prediction model to be validated, we fitted a new lasso regression model to the discovery dataset using the VOCs that were reported to be selected in at least 80% of the folds as the input variables (Table 1). The advantages of lasso regression are that it performs variable selection (which is particularly useful in cases with a large number of features) and reduces overfitting by penalising the model. The performance of the final prediction model was re-estimated through internal validation by LOOCV. The predictive ability of the model was reflected by the corresponding receiver operating characteristics (ROC) curve and the accompanying area under the curve (AUC).

External validation of the prediction model was performed by applying the model to the validation cohort to predict the outcome of the independent samples (patient/control). The cut-off value determined on the discovery cohort was used as the decision threshold. The degree of agreement between the predicted and actual outcome of the participants was determined and expressed in terms of sensitivity, specificity, negative predictive value (NPV), positive predictive value (PPV), and accuracy.

Baseline clinical characteristics were compared within and between the discovery and validation cohort. For continuous variables, the Student t-test or Mann–Whitney U test was performed, after assessing normality. Categorical variables were compared using Fisher’s exact test. For significant variables between both cohorts, Kendall’s τ rank correlation coefficients were calculated to assess a possible association between the variable and the VOCs of the model.

#### 2.3.3. Model Updating

To update the prediction model, a new lasso regression was fitted to the validation cohort using only the predictor variables of the original model as the input features. The predictive performance of the updated model was estimated using LOOCV as the internal validation procedure. As for the original prediction model, a ROC curve was constructed, and the performance characteristics were determined.

## 3. Results

### 3.1. Participant Characteristics

In total, 123 participants were included in the validation study: 47 MPM patients and 76 AEx controls. The clinical characteristics of both the discovery and validation cohort are shown in Table 2. No significant differences between both cohorts could be observed in terms of sex, BMI, smoking status, and packyears. The AEx controls were also similar in age, whereas the MPM patients in the validation cohort were slightly older than those in the discovery cohort (69.99 vs. 66.43 years, respectively, *p* = 0.018). Within both cohorts, the MPM patients were significantly older and had a slightly lower BMI compared with the AEx controls.

### 3.2. Model Validation

**Figure 1:**
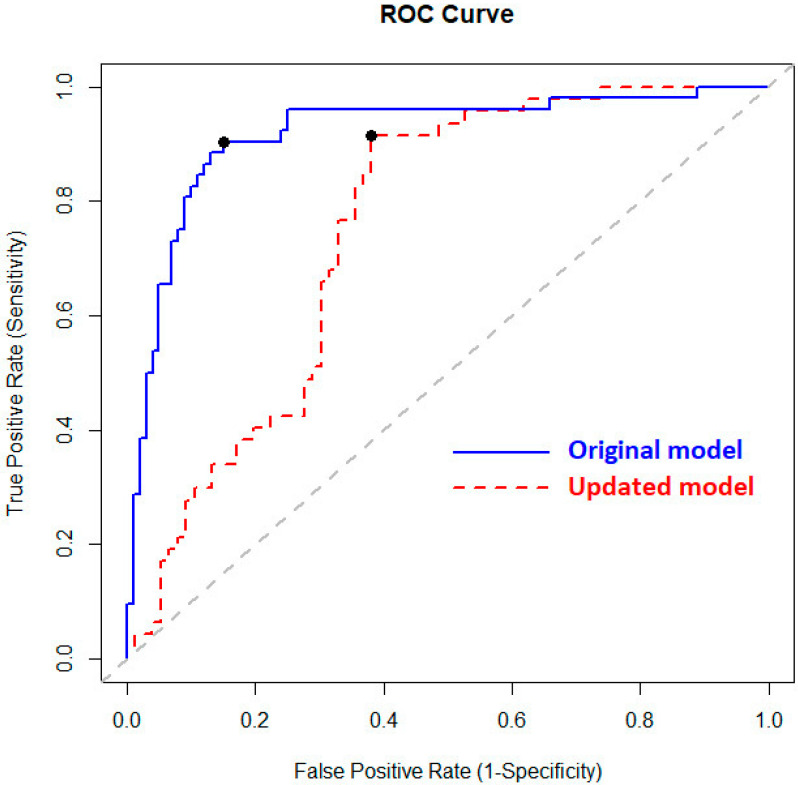
Receiver operating characteristics (ROC) curve of the original (discovery study) and updated (validation study) prediction models, reflecting the models’ predictive ability as estimated by leave-one-out cross-validation. The area under the curve (AUC) is 92% (95% CI: 86–96%) for the original model and 75% (95% CI: 66–83%) for the updated model. The marked points (black dots) correspond to the determined decision thresholds of both models (cut-off value original model: 0.369, cut-off value updated model: 0.358).

The original prediction model, fitted to the discovery cohort, differentiated MPM patients from AEx controls with 87% accuracy, as estimated by LOOCV (Table 3). Eleven VOCs were selected by the lasso regression to be included in this prediction model, which were P1, P7, P9, P15, P21, P26, P84, P88, P101, P122, and P236 (model specifications in Table S1). The corresponding ROC curve is shown in Figure 1, which had an AUC of 92% (95% CI: 86–96%).

Prediction of the participants’ outcome of the external validation samples by this original classification model appeared to be inaccurate as the accuracy was estimated at only 41%. This poor performance was also reflected in the low sensitivity (53%), specificity (33%), PPV (33%), and NPV (53%) values (Table 3). It must thus be stated that the external validation of the original prediction model showed that the latter is not generalisable to the general patient/control population.

As the age of the MPM patients was the only clinical characteristic to significantly differ between the discovery and validation cohort, a correlation analysis was performed to assess any association between this parameter and the VOCs of the prediction model. Of the 11 VOCs, only P15 showed a weak correlation with age (Kendall’s τ = 0.172, *p* = 0.012; Table S2).

In addition, based on the test samples taken with and without an additional viral filter, no significant effect could be observed of the viral filter on the VOCs analysed in this study (Table S3).

### 3.3. Model Updating

To update the original model and to assess the validity of the 11 discriminatory VOCs included in the original model, a new lasso regression was fitted to the external validation cohort using this subset of 11 VOCs as the input variables. The VOCs selected by the lasso and thus included in this updated model were P9, P88, P101, and P122 (model specifications in Table S1). By updating the model, four out of the eleven VOCs of the original model were retained as important predictors (P9, P88, P101, and P122), albeit with re-estimated coefficients, while the other seven VOCs of the original model were considered irrelevant features for the prediction of the outcome and were removed from the model (P1, P7, P15, P21, P26, P84, and P236).

With 73% accuracy, 92% sensitivity, and 62% specificity, the updated model showed an improved performance on the validation samples compared with the original prediction model (Table 3). The corresponding ROC curve is displayed in Figure 1 and had an AUC of 75% (95% CI: 66–83%).

## 4. Discussion

Research in the field of breathomics has led to a plethora of studies associating VOCs with pathological conditions [15,16]. However, the lack of comprehensive validation studies in this research field has caused VOC-based prediction models to be rarely implemented in clinical practice. Considering all the effort that has gone into discovery studies, this could be considered a substantial waste of research resources and time. Most of the studies mainly focus on highlighting the great potential of VOCs as non-invasive biomarkers but often fail to evaluate the performance of the model using independent data, which is key to verifying the model’s applicability to the general patient population [17].

This study aimed to tackle this lack of external validation by performing the first external validation study of a VOC-based prediction model for MPM. To evaluate the reproducibility and generalisability of the model, an independent participant cohort was included several years after the discovery study that reported differentiation of MPM patients from AEx controls with 85% accuracy, as estimated by a LOOCV procedure [12]. After extraction of the final prediction model and re-estimating its performance, we applied this original model to the external validation cohort. This resulted in an important decline in accuracy compared with the internal validation (from 87% to 41%). Such a strong reduction in accuracy is a quite typical phenomenon in external validation studies, as the initial model was designed to optimally fit the discovery cohort and thus potentially suffers from overfitting [18]. Many factors could potentially have contributed to this drop in predictive capacity, such as differences in sampling location, time, or interobserver variability during VOC analysis. Despite the presence of automated peak detection methods, manual peak selection by experts in the field is still the gold standard for the analysis of MCC/IMS data, as this is less prone to overselection of peaks and yields a higher accuracy [19]. However, because of the subjective nature of this manual process and the difference in analysis experts between the discovery and validation study, the risk of interobserver variability inevitably increased, which could have led to a less well fit of the model. As far as potential clinical confounding factors are concerned, the discovery and validation cohort were well balanced for sex, BMI, smoking status, and packyears. Only a small difference in age between the MPM patients of both cohorts could be observed (69.99 vs. 66.43 years). However, the correlation analysis within this patient group revealed that only one of the eleven VOCs of the original model was weakly correlated with age. In addition, age-related effects on breath VOC profiles are not uniformly acknowledged, and the studies that do show any effect typically compared groups with much larger age differences, so it can thus be assumed that this small age difference will not have impacted the results [20,21]. Therefore, the reduced predictive performance should not be attributed to an imbalance in the known clinical characteristics. It can hence be stated that the decreased model performance is probably due to a combination of an unlimited list of potential influencing factors about which one can only speculate, but which certainly require more attention from the breath research community.

External validation showed poor performance of the original model, which is, as mentioned before, a quite typical phenomenon. In practice, these “failing” models are often rejected and replaced by completely new ones. However, this is considered a waste of scientific data from existing studies, which goes against the principle that scientific inferences should be based on as much information as possible [17,22]. A much better approach would be to adjust or update the original model to improve its performance and thereby combine information from the discovery study captured in the original model with information from the new validation cohort [22,23].

Therefore, next to external validation, our study also presents a way of updating the model while integrating information from the discovery study. To do this, a new lasso regression was fitted to the validation cohort using the 11 discriminatory VOCs of the original model of the discovery study as the input variables. This resulted in an updated model that was created by removing seven irrelevant variables and re-estimating the predictor weights (coefficients) of four retained, informative variables compared with the original model. The updated model showed a better performance on the validation samples by discriminating patients and controls with 73% accuracy. With high sensitivity (92%) and NPV (92%) values, the model exhibited the required characteristics of a potential screening test that could allow for ruling out MPM in the asbestos-exposed population [24]. As this model is adjusted to the features of a new cohort, it is also expected to be more generalisable to other individuals of the target population [22]. This approach also gave us the opportunity to assess the validity of the 11 discriminatory VOCs that were selected in the discovery study. As retaining four of these discriminatory VOCs (P9, P88, P101, and P122) and re-estimating their coefficients improved the model’s performance, the added value and usefulness of these four VOCs can be substantiated. Moreover, updating the prediction model even led to model simplification (reduced number of features), which generally improves model interpretability and reduces the risk of overfitting [25]. However, although promising, the updated model in turn requires external validation to validate these findings.

What could be considered as a potential limitation of this study is the fact that we do not know the chemical identity of the VOCs as we opted for validating a prediction model that is based on MCC/IMS data. MCC/IMS is a technique that only allows for “pseudo-identification” of VOCs, returning a list of peaks with unique retention times and ion mobility characteristics. The advantages of MCC/IMS are its high sensitivity and analytical speed, relatively low cost, portability, and ease of use in a clinical setting [26]. These benefits make MCC/IMS an attractive technique for clinical practice, allowing for potential disease diagnosis based on the recognition of peak patterns without the need for further chemical identification [27]. However, to obtain more information about the identity of the VOCs, the MCC/IMS data could be cross-checked against additional gas chromatography–mass spectrometry data [12,28]. An additional point of attention is the sample size of the study. Although few uniform guidelines on sample size considerations are at hand, a current rule-of-thumb is to include a minimum of 100 events in the validation cohort to ensure precise performance estimates, as too small sample sizes may lack sufficient power to detect differences in performance [29,30]. As MPM is a rare disease, this number is challenging to reach, which is why we initially settled for 47 MPM patients. However, in our case, this smaller sample size proved to be sufficient to demonstrate the significant decrease in model performance compared with the discovery study. A final limitation is related to the emergence of the COVID-19 pandemic during this validation study. As a consequence, we had to take additional safety precautions in order not to endanger the participants. Specifically, this meant using an additional viral filter during the sampling procedure. Although the material of the filter, polypropylene, is reported to have no VOC absorption capacity, which we also demonstrated through our test experiments, this is a minor modification to the protocol, which should be considered as a possible factor contributing to the discrepancies between the discovery and validation results [31,32].

## 5. Conclusions

This external validation study was the first one to be performed regarding a VOC-based prediction model for MPM. The results revealed a poor performance of the original model when applied to an independent validation set, demonstrating the issue of reproducibility and generalisability in the breath research field. An approach to improve model performance is updating the model instead of discarding it, which also allowed us to verify the validity of the discriminatory VOCs that were included in the original model of the discovery study. Adjustment of the model led to promising outcomes, but it is now of importance that this updated model is in turn validated externally, ideally by an independent research group. Only in this way can a clinically useful prediction model for MPM be established.

## Figures and Tables

**Table 1:** List of volatile organic compounds (VOCs) selected as important predictor variables in the initial discovery study with their corresponding retention time (RT) and inverse reduced ion mobility (1/K_0_). Data selected from [12].

VOC Peak	RT (s)	1/K_0_ (V·cm^−2^)
P1	5.9	0.503
P7	6.6	0.578
P9	1.6	0.601
P15	4.5	0.715
P21	1.2	0.514
P26	4.2	0.689
P84	116.1	0.742
P88	5.5	0.657
P101	20.0	0.716
P122	8.0	0.610
P151	9.5	0.616
P153	253.3	0.599
P159	273.3	0.594
P161	8.9	0.781
P167	5.6	0.715
P173	146.9	0.658
P178	33.9	0.804
P236	3.6	0.733
P240	151.1	0.772

**Table 2:** Overview of the baseline clinical characteristics of the two participant classes in the discovery and validation cohort.

	MPM Patients			AEx Controls				
	Discovery Cohort	Validation Cohort	*p*-Value	Discovery Cohort	Validation Cohort	*p*-Value	*p*-Value *	*p*-Value **
Subjects	52	47		100	76			
Sex (M/F)								
Male	43	39		98	71			
Female	9	8	1 ^a^	2	5	0.241 ^a^	0.001 ^a^	0.078 ^a^
Age	66.43 ± 8.31	69.99 ± 6.36	0.018 ^b^	55.72 ± 6.62	55.73 ± 9.47	0.991 ^b^	<0.001 ^b^	<0.001 ^b^
BMI	25.29 ± 3.10	25.40 ± 3.56	0.876 ^b^	27.59 ± 3.84	27.25 ± 4.19	0.585 ^b^	<0.001 ^b^	0.010 ^b^
Smoking status								
Never	19	22		34	33			
Current	5	3	0.561 ^a^	22	10	0.235 ^a^	0.160 ^a^	0.575 ^a^
Ex	28	22		44	33			
Packyears	2.65	5.25	0.962 ^c^	5.80	1.50	0.170 ^c^	0.356 ^c^	0.686 ^c^
	(0.00–14.55)	(0.00–20.00)		(0.00–24.15)	(0.00–15.00)			
BARD diagnosis								
Pleural plaques				35	6			
Pleural thickening				2	0			
Asbestosis				3	2			
Pleuritis				1	3			

Values are presented as n, mean ± SD or median (Q1–Q3). AEx: asbestos-exposed; BARD: benign asbestos-related disease; MPM: malignant pleural mesothelioma. ^a^: Fisher’s exact test; ^b^: T-test; ^c^: Mann–Whitney U test. *: comparison of MPM patients vs. AEx controls within the discovery cohort; **: comparison of MPM patients vs. AEx controls within the validation cohort.

**Table 3:** Performance characteristics of the original prediction model, determined by internal and external validation, and of the updated prediction model, determined by internal validation.

	Original Model		Updated Model
	Internal Validation (Study 2017)	External Validation (Study 2022)	Internal Validation (Study 2022)
Sensitivity	90.4 (80.0–96.4)	53.2 (39.0–67.0)	91.5 (80.8–97.2)
Specificity	85.0 (77.0–91.0)	32.9 (23.1–44.0)	61.8 (50.6–72.2)
PPV	75.8 (64.1–85.2)	32.9 (23.1–44.0)	59.7 (48.2–70.6)
NPV	94.4 (88.2–97.9)	53.2 (39.0–67.0)	92.2 (82.2–97.4)
Accuracy	86.8 (80.8–91.5)	40.7 (32.3–49.5)	73.2 (64.9–80.4)

Values are presented as percentages with their 95% confidence interval. NPV: negative predictive value; PPV: positive predictive value.

## Data Availability

The data presented in this study are available upon request from the corresponding author.

## Supplementary Materials

The following supporting information can be downloaded at: https://www.mdpi.com/article/10.3390/cancers14133182/s1. Table S1: Model Specifications of the original and updated prediction models. Table S2: Kendall’s τ rank correlation between the age of the pleural mesothelioma patients and the volatile organic compounds (VOCs) of the original model. Table S3. Comparison of the peak intensities of the analysed volatile organic compounds between breath samples taken with (*n* = 3) and without (*n* = 3) additional viral filter (paired Wilcoxon signed rank test).
