# Supplementary material for: External Validation of a Breath-Based Prediction Model for Malignant Pleural Mesothelioma
Source: Cancers (Basel). 2022 Jun 29;14(13):3182. doi: 10.3390/cancers14133182 (PMC9264774; doi:10.3390/cancers14133182)
Supplement: Supplementary file 1 [file cancers-14-03182-s001.zip › cancers-1755790-supplementary.pdf]

cancers

MDPI

Article

External Validation of a Breath-Based Prediction Model for Malignant Pleural Mesothelioma

Eline Janssens, Eline Schillebeeckx, Kathleen Zwijsen, Jo Raskin, Joris Van Cleemput, Veerle F Surmont, Kristiaan Nackaerts, Elly Marcq, Jan P. van Meerbeeck and Kevin Lamote

Table S1. Model specifications of the original and updated prediction models.

	Original model	Updated model
**Intercept**	0.2863634	-0.01539101
**P1**	-9.3875710	.
**P7**	-3.9149386	.
**P9**	30.9184867	-20.26094596
**P15**	-14.4468673	.
**P21**	-10.1389553	.
**P26**	-30.1241610	.
**P84**	91.8151273	.
**P88**	-19.0494276	34.43419887
**P101**	47.9473706	99.13002708
**P122**	8.2431091	33.61673132
**P236**	0.8623499	.

Model intercept and regression coefficients of the selected features are displayed.

Table S2. Kendall's τ rank correlation between the age of the pleural mesothelioma patients and the volatile organic compounds (VOCs) of the original model.

VOCs	Age	
	Kendall's τ	p-value
**P1**	0.053	0.435
**P7**	- 0.045	0.506
**P9**	- 0.051	0.453
**P15**	0.172	0.012*
**P21**	0.112	0.102
**P26**	0.040	0.560
**P84**	- 0.060	0.385
**P88**	0	1
**P101**	0.049	0.479
**P122**	- 0.026	0.710
**P236**	- 0.094	0.168

*p < 0.05.

Table S3. Comparison of the peak intensities of the analysed volatile organic compounds between breath samples taken with (*n* = 3) and without (*n* = 3) additional viral filter (paired Wilcoxon signed rank test).

Peak	Peak intensity		p-value
	With viral filter	Without viral filter	
**P1**	0.0483 (0.0476-0.0500)	0.0543 (0.0513-0.0563)	0.438
**P7**	0.0250 (0.0249-0.0250)	0.0219 (0.0195-0.0235)	0.400
**P9**	0.0095 (0.0088-0.0103)	0.0107 (0.0104-0.0109)	0.438

**P15**	0.0125 (0.0122-0.0127)	0.0142 (0.0127-0.0144)	0.438
**P21**	0.0047 (0.0045-0.0051)	0.0054 (0.0052-0.0060)	0.438
**P26**	0.2085 (0.1940-0.2250)	0.1363 (0.1160-0.2010)	0.438
**P84**	0.0004 (0.0002-0.0005)	0.0000 (0.0000-0.0003)	0.201
**P88**	0.0092 (0.0092-0.0097)	0.0096 (0.0090-0.0100)	0.400
**P101**	0.0020 (0.0019-0.0021)	0.0011 (0.0010-0.0012)	0.438
**P122**	0.0131 (0.0119-0.0135)	0.0135 (0.0116-0.0138)	0.438
**P236**	0.0264 (0.0246-0.0300)	0.0180 (0.0174-0.0254)	0.438

Values presented as median (Q1-Q3).
